# Endurance-Oriented Model Predictive Energy Management for a Proton Exchange Membrane Fuel Cell–Battery Hybrid Quadcopter Under Dynamic Mission Conditions

**DOI:** 10.3390/ma19122548

**Published:** 2026-06-12

**Authors:** Murat Kayaoğlu, Sencer Ünal, Hilal Biyik

**Affiliations:** Department of Electrical and Electronics Engineering, Institute of Science, Engineering Faculty, Firat University, 23000 Elazig, Türkiye; sencerunal@firat.edu.tr (S.Ü.); hbiyik@firat.edu.tr (H.B.)

**Keywords:** proton exchange membrane fuel cell, hybrid UAV, energy management strategies, endurance optimization

## Abstract

Proton exchange membrane fuel cell–battery hybrid power systems provide an effective solution to overcome the limited endurance of battery-powered multirotor unmanned aerial vehicles. However, the highly transient power demands of quadcopter platforms, combined with balance-of-plant losses and operational constraints, create significant challenges for reliable energy management. This study proposes a degradation-aware stress-mitigation model predictive control-based energy management framework to maximize mission endurance under realistic conditions. A control-oriented, physics-consistent model is developed using manufacturer polarization data from a 500 W Aerostak proton exchange membrane fuel cell. The model captures polarization behavior, balance-of-plant loads, battery dynamics, and direct current-bus power balance. The model predictive control strategy optimally allocates power by maintaining direct current-bus stability, regulating battery state-of-charge within safe limits, and constraining fuel cell power ramp rates to mitigate degradation. High-fidelity simulations are conducted under stochastic wind disturbances and mission-dependent load profiles, including takeoff, climb, cruise, and maneuvering phases. The results show continuous power delivery without unmet load demand. The hybrid system achieves a flight endurance of 220–224 min, consuming a total of 89.99 g of hydrogen at an average rate of 0.398–0.412 g/min, indicating a notable reduction under the considered operating conditions. Additionally, long-term analysis indicates that over 97% of initial endurance is preserved after 100 cycles, demonstrating robustness against fuel cell aging. An analytical real-time feasibility assessment further indicates that the control-oriented formulation is compatible with the computational resources of typical unmanned aerial vehicle-class onboard processors, while the integration of adaptive and robust predictive control techniques is identified as a direction for future work.

## 1. Introduction

Unmanned aerial vehicles (UAVs), particularly quadcopter platforms, have become indispensable tools in a wide range of civilian and industrial applications, including surveillance, mapping, search-and-rescue operations, and infrastructure inspection. Their ability to hover, maneuver precisely in confined environments, and perform vertical takeoff and landing makes quadcopters especially attractive for complex missions. However, despite these operational advantages, the flight endurance of multi-rotor UAVs remains one of the most critical limitations, primarily due to the constraints imposed by onboard energy storage systems [1,2,3].

Conventional lithium-ion and lithium-polymer battery-based architectures provide high power density and fast dynamic response, which are well suited for the transient power demands of quadcopters. Nevertheless, their relatively low specific energy significantly restricts achievable flight duration, particularly for long-endurance or energy-intensive missions. As UAV deployment continues to expand into applications requiring extended operational times and reduced environmental impact, the development of alternative energy sources with higher energy density, along with advanced energy management strategies, has become a central research challenge in aerial robotics [4,5,6].

Hydrogen has recently attracted considerable attention as a promising next-generation energy carrier due to its high gravimetric energy density and environmentally sustainable energy conversion characteristics. Unlike conventional fossil fuels, hydrogen-based systems enable electrochemical energy conversion with near-zero local emissions while offering higher specific energy compared to conventional lithium-based battery technologies. In addition, hydrogen energy systems provide an effective solution for energy storage and conversion, particularly in applications where long operational duration and energy sustainability are critical. These physicochemical advantages have positioned proton exchange membrane fuel cells (PEMFCs) as one of the most promising power generation technologies for UAV platforms requiring extended endurance, stable power delivery, and reduced environmental impact [7].

PEMFCs offer several attractive characteristics for aerial platforms, including low operating temperature, rapid start-up capability, quiet operation, and environmentally sustainable electrochemical energy conversion [8,9,10]. Unlike combustion-based power systems, PEMFCs generate electrical energy through an electrochemical process that produces only water as a byproduct. This feature not only reduces environmental impact but also enables quiet operation, which is particularly advantageous in surveillance, reconnaissance, and urban UAV missions [11,12]. Previous studies have consistently demonstrated that PEMFC-based hybrid power architectures can significantly enhance UAV flight endurance compared with battery-only systems. Reported results indicate notable endurance improvements in rotary-wing platforms and, in some cases, multi-fold endurance increases in fixed-wing UAV configurations, primarily due to the superior gravimetric energy density of hydrogen-based energy storage systems [11].

Despite their clear advantages in terms of energy density and sustainability, PEMFCs exhibit inherent limitations when subjected to highly dynamic load profiles. In quadcopter UAVs, power demand can change rapidly during takeoff, climbing, aggressive maneuvering, and disturbance rejection, placing stringent requirements on the power source’s transient response. The relatively slow dynamic behavior of PEMFCs, combined with the power consumption of auxiliary balance-of-plant (BoP) components such as air supply and thermal management systems, makes it challenging for fuel cells to independently guarantee power continuity under such conditions [13,14]. Consequently, PEMFCs are most effectively employed in hybrid configurations, where they are coupled with fast-response secondary energy storage devices, such as batteries or supercapacitors. In these hybrid architectures, the PEMFC typically supplies the average or low-frequency power component, while the secondary storage compensates for transient power demands, thereby ensuring stable operation and protecting the fuel cell from excessive load fluctuations [15,16].

While PEMFC–battery hybridization offers significant potential for extending flight endurance, it introduces substantial complexity in energy management. During flight, the UAV power demand varies continuously as a function of mission phase, environmental disturbances (e.g., wind), maneuver intensity, and vehicle dynamics. As a result, the power-sharing strategy between the fuel cell and the battery must be optimized in real time while satisfying multiple constraints, including fuel cell power limits, battery state-of-charge (SoC) bounds, and overall system efficiency requirements [17]. Traditional rule-based energy management strategies can be effective under predefined operating conditions; however, their reliance on fixed heuristics and extensive parameter tuning limits adaptability and often leads to suboptimal performance under varying and uncertain flight profiles [18,19].

In this context, Model Predictive Control (MPC) has gained increasing attention as an advanced energy management framework for hybrid power systems. MPC exploits an explicit model of system dynamics and a finite prediction horizon to optimize control actions while directly enforcing operational constraints. For PEMFC-based hybrid systems, MPC enables simultaneous optimization of multiple objectives, including hydrogen consumption minimization, battery SoC regulation, constraint satisfaction, and mitigation of component stress [15,20,21]. By maintaining the fuel cell operation within favorable efficiency regions and actively managing the battery SoC around a desired reference level, MPC-based strategies can improve both energy efficiency and system durability [15,22]. Furthermore, when combined with load or trajectory prediction and data-driven forecasting techniques, MPC has been shown to enhance robustness against disturbances and modeling uncertainties, which is particularly relevant for UAV applications operating in dynamic environments [23,24].

In this study, an MPC-based energy management framework is proposed for a PEMFC–battery hybrid quadcopter power architecture with the primary objective of extending flight endurance under realistic operating conditions. The controller optimizes power flows along the entire flight profile by explicitly incorporating system dynamics and operational constraints into the decision-making process. Through this formulation, hydrogen consumption is managed efficiently, the battery state of charge is regulated within safe and functional limits, and power continuity is preserved during transient load events, thereby enabling sustained and reliable long-endurance missions.

Beyond system-level energy management, the operational performance, durability, and efficiency of PEMFC-powered UAV systems are strongly influenced by electrochemical, thermal, and material-sensitive phenomena occurring within the fuel cell stack and auxiliary subsystems. In particular, membrane stress, catalyst degradation, thermal regulation, and transient electrochemical loading directly affect long-term operational stability and hydrogen utilization behavior. Accordingly, the present study is not only relevant from a control-system perspective, but also from the standpoint of material-sensitive electrochemical energy system operation and degradation-aware management of PEMFC-based hybrid power architectures. In parallel with such control-oriented perspectives, recent research has advanced data-driven methods for predicting PEMFC performance degradation and quantifying long-term durability. For instance, Transformer-based prediction models have been developed to forecast fuel cell performance degradation while explicitly handling reversible voltage-recovery effects [25], and hybrid frameworks coupling data-driven techniques with electrochemical impedance information have been proposed for state-of-health estimation and degradation quantification of PEMFCs [26]. These approaches represent the current frontier of degradation prediction and provide a promising basis for future integration of explicit lifetime-prediction and health-estimation modules into degradation-aware energy management architectures such as the one considered in this study.

The main contributions of this study are summarized as follows:

- An integrated MPC-based energy management framework is developed for a PEMFC–battery hybrid quadcopter operating under dynamically varying mission conditions.
- A physics-consistent hybrid power system model is established by jointly considering PEMFC electrochemical behavior, Lithium Polymer (Li-Po) battery dynamics, BoP losses, converter-related effects, and propulsion power demand within a unified predictive framework.
- An endurance-oriented predictive control formulation is proposed to simultaneously regulate hydrogen utilization, battery state-of-charge constraints, power continuity, and degradation-related operational stress during transient UAV operation.
- Stochastic disturbance scenarios representing realistic mission variability are incorporated to evaluate robustness of the proposed framework under dynamically changing load conditions.
- A comprehensive system-level performance assessment is conducted in terms of hydrogen consumption, endurance behavior, power continuity, and operational robustness for PEMFC-powered multirotor UAV applications.

## 2. Literature Review

PEMFC-based power systems have attracted significant attention in UAV applications, particularly in missions requiring extended operational endurance. Compared to conventional lithium-ion or lithium-polymer battery systems, PEMFCs offer substantially higher specific energy, making them promising candidates for long-endurance aerial platforms [11]. Previous experimental and system-level studies on fixed-wing UAVs have demonstrated considerable endurance improvements through PEMFC integration, primarily due to the superior gravimetric energy density of hydrogen-based energy storage systems [11]. Similar endurance enhancements have also been reported for rotary-wing and mini-UAV platforms operating under practical flight conditions [12].

Despite these advantages, PEMFC systems exhibit several intrinsic limitations, most notably relatively slow dynamic response characteristics and the presence of auxiliary BoP subsystems such as air compressors, humidification units, and cooling components [9,14]. These limitations become particularly critical in rotor-based UAV applications characterized by highly transient and rapidly varying propulsion loads. Rapid power fluctuations during takeoff, climb, maneuvering, and disturbance rejection cannot be reliably supported by PEMFCs alone. Consequently, hybrid power architectures combining PEMFCs with secondary energy storage elements such as batteries or supercapacitors have become a widely adopted solution [13]. In such hybrid configurations, the secondary storage unit compensates for high-frequency transient power demands, while the PEMFC supplies the average or low-frequency load component, enabling operation closer to its high-efficiency operating region [13]. This coordinated operation not only improves power continuity and transient response capability but also helps reduce degradation-related stress caused by aggressive load oscillations in fuel cell systems, thereby contributing to improved operational durability [15,16].

The introduction of hybrid power architectures, however, substantially increases the complexity of energy management and supervisory control. UAV propulsion demand continuously varies depending on flight phase, maneuver intensity, payload conditions, and environmental disturbances such as wind, requiring real-time coordination between the PEMFC subsystem and the secondary energy storage unit. Early studies predominantly relied on rule-based and frequency-splitting energy management strategies because of their relatively low computational burden and implementation simplicity [13,18]. Although such methods can provide acceptable performance under predefined operating conditions, they generally require extensive parameter tuning and exhibit limited adaptability under rapidly changing mission profiles and uncertain operating environments [17,19].

MPC has emerged as one of the most promising advanced control strategies for energy management in hybrid power systems because of its capability to explicitly incorporate system dynamics, operational constraints, and multi-objective optimization within a unified predictive framework. In PEMFC–battery hybrid systems, MPC-based approaches have demonstrated significant potential for reducing hydrogen consumption, regulating battery SoC, and mitigating operational stress by maintaining the fuel cell within favorable efficiency operating regions [15,21,22]. In addition, the adoption of control-oriented battery models has enabled real-time applicability of MPC implementations while preserving acceptable estimation accuracy, even under relatively long prediction horizons [20]. These characteristics make MPC particularly attractive for UAV applications where efficiency, operational robustness, and constraint satisfaction must be simultaneously considered.

In recent years, MPC-based energy management strategies have increasingly been investigated in UAV applications together with mission-aware and predictive load-management concepts. Existing studies have shown that incorporating predicted mission profiles and anticipated power-demand variations into the predictive layer can improve hybrid power allocation efficiency and increase robustness against external disturbances [23,24,27]. However, despite these advances, the performance of MPC-based frameworks in rotary-wing UAVs remains strongly dependent on the fidelity of the underlying subsystem models, including fuel cell efficiency behavior, battery dynamics, BoP consumption, converter losses, and transient load characteristics [27,28]. Furthermore, practical implementation of predictive control architectures requires a careful balance between prediction horizon length, computational burden, and real-time execution feasibility, particularly for embedded UAV-class onboard processors.

Beyond power allocation alone, recent research has emphasized the importance of integrating energy management with higher-level flight-control and mission-planning layers. Coordinated architectures coupling trajectory tracking and predictive energy management have demonstrated the capability to simultaneously reduce tracking errors and improve overall mission endurance [29]. Similarly, mission-aware planning frameworks capable of generating reference power trajectories for the supervisory control layer have been shown to further improve endurance-oriented operation and system-level efficiency [30]. These findings collectively indicate that predictive and mission-aware control architectures can provide substantial advantages over decoupled or purely heuristic energy management approaches.

Thermal management and environmental adaptability represent additional critical factors influencing PEMFC efficiency, reliability, and long-term operational stability. Previous studies have demonstrated that PEMFC performance is highly sensitive to stack temperature, pressure regulation, humidification behavior, and auxiliary subsystem operation [31]. MPC-based thermal management approaches have shown promising capability in maintaining stack temperature within safe operational limits while simultaneously improving efficiency and operational reliability [32]. Moreover, detailed electro-thermal modeling frameworks have been reported to improve the physical consistency and predictive fidelity of simulation-based hybrid UAV analyses, highlighting the importance of comprehensive multi-domain system modeling in PEMFC-powered aerial platforms [33,34].

Research Gap: Although the existing literature clearly demonstrates the potential of PEMFC-based hybrid UAV architectures for extending flight endurance [11,12], several important limitations remain insufficiently addressed, particularly for rotary-wing UAV platforms operating under highly transient mission conditions. In many previously reported studies, realistic system-level power accounting incorporating BoP losses and power electronic converter losses, endurance-oriented MPC formulations considering both hydrogen utilization and battery operational constraints, disturbance-sensitive mission operation, and comprehensive assessment of power continuity across multiple flight phases are generally investigated separately rather than within a unified predictive energy management framework [15,20,29,30]. Furthermore, relatively limited attention has been devoted to the combined interaction between stochastic mission disturbances, endurance-oriented predictive control, degradation-related operational stress mitigation, and system-level robustness in PEMFC–battery hybrid multirotor UAVs. Motivated by these limitations, the present study proposes an integrated MPC-based energy management framework specifically developed for a PEMFC–battery hybrid quadcopter, where hydrogen utilization, power continuity, operational constraints, and endurance-oriented system performance are jointly evaluated under dynamically varying mission conditions.

## 3. System Architecture and Modeling

The proposed hybrid UAV energy management framework is developed as an integrated multi-domain system in which the PEMFC subsystem, Li-Po battery model, propulsion load dynamics, and MPC-based supervisory controller interact through a common direct current (DC)-bus architecture. Rather than operating as isolated component-level models, the individual subsystems are dynamically coupled through power balance, state evolution, and operational constraints. In this framework, the propulsion load profile and stochastic disturbances determine the instantaneous power demand, while the MPC layer coordinates power allocation between the PEMFC and the battery based on predicted system states, operational limits, and endurance-oriented objectives. Accordingly, the overall formulation should be interpreted as a unified system-level energy management architecture integrating electrical, electrochemical, and control-oriented dynamics within a single predictive framework.

### 3.1. Hybrid Power System Architecture

The proposed quadrotor platform employs a hybrid electric powertrain in which a PEMFC stack serves as the primary energy source, while a Li-Po battery pack acts as a fast-response auxiliary buffer. Both sources are interfaced through a common DC bus that supplies the propulsion (ESC–motor–propeller set) and onboard avionics [5,33,34]. This topology is widely adopted in fuel-cell UAV systems because it decouples the slow fuel-cell dynamics from highly transient rotorcraft power demands and enables constraint-aware power sharing [35,36,37].

Let $P_{load}(k)$ denote the electrical load power demanded by the quadrotor at discrete time step *k*. The DC-bus power balance can be expressed as

(1) $$P_{bus}\left(k\right)=P_{FC,net}\left(k\right)+P_{bat}(k)$$

where $P_{FC,net}(k)$ is the net PEMFC system power delivered to the DC bus after auxiliary loads, and $P_{bat}\left(k\right)\;$ is the battery power (positive in discharge and negative in charge). Any residual mismatch can be represented as an unmet-power term for feasibility diagnostics [37,38,39]:

(2) $$P_{unmet}\left(k\right)=max(0,P_{load}\left(k\right)-P_{bus}\left(k\right))$$

In the proposed Energy Management System (EMS) design, the controller aims to keep $P_{unmet}(k)$ ≈ 0 by allocating power between sources under operational constraints.

#### Mass Budget and Balance of Plant

In aerial robotics, the gravimetric power density of the onboard energy source is a primary determinant of flight endurance. Unlike battery-only systems, where the majority of the system mass is concentrated in the electrochemical cells, fuel cell-based powertrains require additional auxiliary components collectively referred to as the BoP to ensure safe hydrogen storage, fuel delivery, thermal regulation, and power conditioning [40]. Neglecting the mass contribution of these subsystems often leads to overly optimistic endurance estimations in simulation-based UAV studies.

To ensure a physically realizable and conservative system design, this study incorporates a detailed mass budget derived from a representative 500 W-class air-cooled PEMFC stack and a practical hydrogen storage subsystem. As summarized in Table 1, the total maximum take-off mass (MTOM) of the hybrid quadcopter is 5.766 kg, accounting for all propulsion, energy, and avionics components. In the updated configuration, the hydrogen storage system including a 3 L, 400 bar composite tank, pressure regulation hardware, valves, and associated tubing contributes approximately 1.75 kg to the total mass. This reduction relative to earlier configurations reflects an optimized BoP layout while still satisfying safety and regulatory constraints for compressed hydrogen storage in aerial platforms.

Similarly, the airframe mass has been reduced to 950 g through the adoption of a lightweight carbon fiber quadrotor structure [41], further improving the overall gravimetric efficiency of the system [42,43]. Despite these reductions, the BoP associated with hydrogen storage remains the single largest mass contributor (30.3% of Maximum Take-Off Mass (MTOM)), highlighting the inherent trade-off between system-level safety requirements and endurance gains in hydrogen-powered UAVs. The resulting mass budget is used consistently in the dynamic load calculations and power demand profiles employed within the MPC-based energy management simulations, ensuring that the reported endurance and performance results remain physically grounded and representative of a realizable hybrid UAV platform [42,43,44].

### 3.2. PEMFC System Model

This subsection presents the mathematical modeling framework of the PEMFC subsystem employed in the proposed hybrid UAV power architecture. The model is developed to capture the electrochemical behavior, power-generation characteristics, auxiliary subsystem losses, and hydrogen consumption dynamics relevant to endurance-oriented UAV operation. To improve the physical consistency of the simulation framework, the PEMFC representation includes voltage polarization effects, BoP power consumption, and hydrogen utilization behavior under dynamically varying load conditions. The following subsections describe the fuel cell voltage characteristics, net system power formulation, and hydrogen consumption model integrated into the MPC-based energy management framework.

#### 3.2.1. Fuel Cell Voltage and Polarization Losses

The PEMFC stack is modeled as a controllable current source whose output voltage is governed by the load current and the associated electrochemical loss mechanisms. To ensure physically consistent behavior over the entire operating range, the polarization characteristics of the stack are represented by explicitly accounting for activation, ohmic, and concentration (mass transport) losses, which is a standard practice in control-oriented PEMFC modeling studies [29,33].

Under rapidly varying load conditions, however, the PEMFC output voltage cannot instantaneously follow the demanded current because of electrochemical reaction kinetics, gas transport delays, and double-layer capacitance effects. These transient phenomena include electrochemical charge-transfer dynamics, thermal behavior of the fuel cell stack, and anode/cathode flow-channel dynamics, all of which may cause noticeable fluctuations in cell voltage, temperature, and reactant flow rates during abrupt load variations. In particular, the combined effect of double-layer capacitance and charge-transfer resistance determines the effective transient response characteristics of the PEMFC system, leading to operating-condition-dependent response times.

Although detailed electrochemical transient models can provide high modeling accuracy, they generally introduce substantial computational complexity, which limits their applicability in real-time MPC-based energy management implementations. Furthermore, PEMFC systems inherently exhibit strong nonlinearities, coupled multi-physics interactions, and operating-condition-sensitive dynamic behavior, making high-fidelity electrochemical representation computationally demanding for predictive control applications. Therefore, the present study adopts a control-oriented and physics-consistent semi-empirical PEMFC formulation that preserves the dominant polarization and transient behavior while maintaining computational efficiency suitable for system-level predictive energy management analysis [45,46,47].

To approximately represent the behavior of a PEMFC under sudden load changes, four main models are used in the literature. The most common is a control-oriented approach, in which the double-layer capacitance and gas transport delays are all represented by a single effective time constant.

The transient voltage dynamics are expressed as shown in Equation (3).

(3) $$\tau_{FC}\frac{{dV}_{FC}(t)}{dt}+V_{FC}\left(t\right)=V_{ss}(t)$$

Here, $V_{FC}\left(t\right)$ represents the transient fuel cell output voltage, $V_{ss}(t)$ denotes the steady-state voltage obtained from the semi-empirical polarization model, and $\tau_{fc}$ is the effective PEMFC time constant representing the electrochemical and gas transport dynamics. Thanks to this simplified dynamic structure, the MPC algorithm can represent the dominant transient voltage recovery behavior during rapid power demand changes while maintaining real-time computational efficiency. Furthermore, the transfer function form is given in Equation (4).

(4) $$\frac{V_{FC}\left(s\right)}{V_{ss}(t)}=\frac{1}{\tau_{FC}s+1}$$

Here, the time constant is $\tau_{FC}=R_{d}{\times C}_{dl}$, where $R_{d}$ is the dynamic resistance and $C_{dl}$ is the double-layer capacitance.

This model has been experimentally validated to show good agreement with measurement data, representing the double-layer charging effect over short time intervals (below 1 s) as well as the fuel and oxidant flow delays and thermodynamic characteristics over long time intervals (several minutes) [48].

Another model represents the voltage response to sudden load changes as the sum of three exponential functions, as shown in Equation (5) [49].

(5) VFCt=Vss+A1e−tτ1+A2e−tτ2+A3e−tτ3

Here,$\tau_{1},\tau_{2},\tau_{3}$ represent the different time constants corresponding to the electrochemical, water transport, and heat transfer processes, respectively, while $A_{1},\;A_{2},\;A_{3}$ denote the amplitude factors obtained through correlation from steady-state operating data (temperature, humidity, current, resistance, voltage). Each of the three terms in Equation (5) is an exponential (base-e) decay function of the form Aie−tτi, rather than a power-law expression; the amplitude factors $A_{i}$ multiply the exponential terms, while the time constants $\tau_{i}\;$ appear in the exponent.

The transient evolution of the voltage is modeled as the sum of three exponential functions; the resulting time constants reflect the membrane heat and water transport processes. These model parameters form the basis for estimating voltage overshoot/undershoot in computation-based control systems and real-time simulation [49].

The simple dynamic model, on the other hand, proposes a practical model that combines the steady-state polarization model with first-order thermal dynamics.

(6) $$V_{FC}\left(t\right)=V_{ss}\left(I_{FC}\right)-{∆V}_{act}\left(t\right)-{∆V}_{conc}(t)$$

(7) $$\tau_{th}\frac{{dT}_{st}}{dt}={Q˙}_{gen}-{Q˙}_{cool}$$

Transient voltage deviation with exponential recovery

(8) ∆Vt=∆Vo.e−tτFC

The commercial 1.2 kW Nexa PEMFC power module was operated under various steady-state and transient load current schedules; the relatively good agreement between the measured and predicted output voltage histories demonstrated the practical applicability of the proposed simple dynamic model [50].

Accordingly, the stack voltage is expressed in a semi-empirical form as [51]:

(9) $$V_{FC}\left(k\right)=N_{cell}(E_{Nerst}-V_{act}\left(I_{FC}\right)-V_{ohm}\left(I_{FC}\right)-V_{conc}\left(I_{FC}\right))$$

where $N_{cell}$ denotes the number of cells in the stack and $I_{FC}$ represents the stack current. This formulation captures the dominant electrochemical phenomena governing PEMFC behavior and enables an accurate description of voltage decay as the operating current increases. The adopted structure is consistent with widely reported PEMFC modeling approaches and provides a suitable balance between physical fidelity and computational efficiency for system-level simulations [51,52].

**Figure 1:**
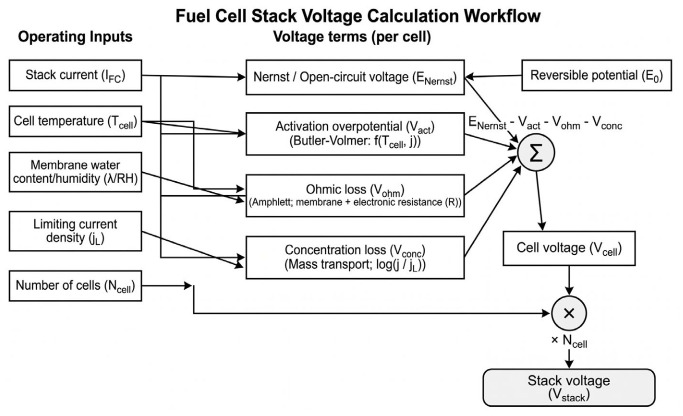
Block diagram of the fuel-cell voltage computation module. The operating inputs feed the open-circuit (Nernst) voltage and the activation, ohmic, and concentration loss terms, which are combined per cell and scaled by the number of cells to yield the stack voltage $V_{FC}$ (Equation (9)).

The structure of the fuel-cell voltage computation module is summarized in Figure 1. The module takes the stack current, cell temperature, membrane water content, limiting current density, and the number of cells as inputs, evaluates the open-circuit (Nernst) voltage and the activation, ohmic, and concentration loss terms on a per-cell basis, combines them, and scales the result by the number of cells to obtain the stack voltage (Equation (9)).

In this study, the voltage losses of the PEMFC were not only expressed by a general polarization equation, but the behavior of each loss component depending on the fuel cell operating variables was also parametrically modeled. The PEMFC output voltage was calculated by considering the combined effect of the open-circuit voltage, activation loss, ohmic loss, and concentration loss components. Based on the semi-empirical polarization model proposed by Santarelli et al., the activation losses were modeled using a Butler–Volmer-based approach, and it was assumed that these losses vary depending particularly on the cell temperature and current density. Accordingly, the cathode exchange current density, $i_{oc}$, increases as the temperature rises, and consequently the activation overvoltage decreases. Ohmic losses, on the other hand, were considered as the sum of the membrane ionic resistance and electronic resistance components. It was taken into account that the membrane ionic resistance varies depending on operating variables such as cell temperature, membrane humidity, membrane thickness, and operating current. For this purpose, based on the Amphlett model, the ionic resistance was defined as a function of temperature and current density. In particular, it was modeled that as the membrane water content increases, the proton conductivity rises and the ohmic losses decrease. Concentration losses were modeled based on mass transport limitations occurring at high current densities. In this context, using the limiting current density (i_l_), the voltage drops due to reduced reactant diffusion were calculated using a logarithmic expression. Thus, the effects of oxygen and hydrogen transport limitations under high-load conditions were incorporated into the model. Moreover, the model parameters were calibrated using manufacturer polarization curves and experimental study data; the effects of operating variables such as temperature, current density, and membrane humidity on the PEMFC voltage behavior were determined using a regression-based parameter estimation method. Consequently, the developed PEMFC model was constructed to represent not only the theoretical polarization equation but also the dynamic voltage losses dependent on actual operating conditions [53].

The model parameters were calibrated against the manufacturer’s polarization data of the Aerostak A-500 (500 W) air-cooled PEMFC stack to ensure realistic voltage–current behavior [54]. As demonstrated in the model validation results, the simulated polarization curve closely follows the datasheet characteristics across the nominal operating range, confirming that the activation and ohmic loss components are accurately represented. Minor deviations observed at very low current levels can be attributed to open-circuit voltage approximation and auxiliary stack losses, which are not explicitly specified in the manufacturer documentation [52,55].

**Figure 2:**
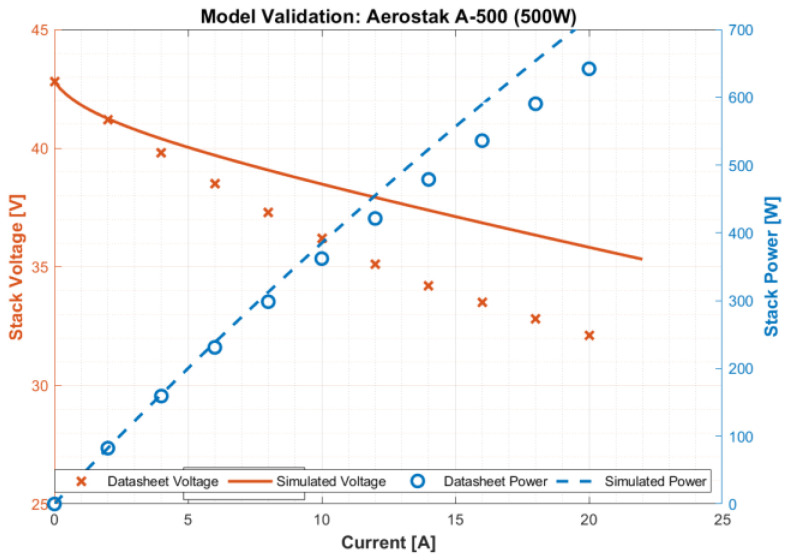
Validation of the PEMFC electrochemical model: Comparison between the Aerostak A-500 manufacturer data (markers) and the simulation results (lines) for both voltage and power characteristics.

Figure 2 compares the manufacturer’s reference data points with the output of the simulated model implemented in this study. Rather than summarizing the agreement with a single error metric over the full current range, the model accuracy is characterized by operating region. In the low-to-medium current region, which corresponds to the region in which the fuel cell is actually operated in the proposed hybrid configuration, the simulated voltage profile follows the manufacturer polarization characteristic closely. At higher currents, however, the deviation increases, reaching approximately 12.5% near 20 A. This validation ensures that the subsequent energy management and endurance analyses, which rely on the low-to-medium current region, are based on realistic power generation dynamics.

The PEMFC polarization model was implemented in the MATLAB/Simulink R2026a environment using a semi-empirical formulation that includes the activation, ohmic, and concentration overvoltage components. The model parameters were calibrated in the 0–20 A operating range using the manufacturer polarization characteristics of the Aerostak A-500 PEMFC stack. The validation results showed high agreement between the simulation curves and the polarization curves provided by the manufacturer in the low and medium current operating regions. However, as shown in Figure 2, the deviation between the simulation and reference curves becomes more pronounced at high current levels, reaching approximately 12.5% around 20 A. The main reason for this increasing mismatch is the nonlinear mass transport effects, membrane humidification dynamics, and concentration-related losses that are not fully represented in the simplified control-oriented polarization model. Accordingly, the agreement should be interpreted on a region-specific basis: the model is accurate in the low-to-medium current region, in which the fuel cell is operated within the proposed hybrid configuration, whereas the larger high-current deviation lies outside the fuel cell’s actual operating envelope, since the battery supplies the high-current transient peaks. In this context, the proposed PEMFC model aims to offer a physically consistent approach with computational efficiency suitable for system-level energy management and MPC-based control analyses, rather than a detailed electrochemical model that represents all internal electrochemical dynamics with high accuracy.

To account for transient effects under rapid load variations, a simplified dynamic voltage component may be incorporated, for instance through an equivalent RC term representing double-layer charging and diffusion-related delays [51,52]. Such a time-constant-based transient representation is consistent with reported PEMFC dynamic behavior and provides a practical compromise between modeling accuracy and computational tractability, which is particularly important for real-time MPC-based energy management applications [36,56].

#### 3.2.2. Net Fuel Cell System Power and BoP Loads

The gross fuel cell electrical power is

(10) $$P_{FC}\left(k\right)=V_{FC}(k)I_{FC}(k)$$

The net power delivered to the DC bus explicitly accounts for BoP auxiliary loads (e.g., air supply and thermal management), expressed as:

(11) $$P_{FC,net}\left(k\right)=P_{FC}\left(k\right)-P_{aux}(k)$$

Including $P_{aux}$ is essential for realistic endurance estimation and for preventing optimistic hydrogen-to-electricity conversion assumptions in UAV-scale PEMFC systems [5,42,44].

#### 3.2.3. Hydrogen Consumption

Hydrogen mass consumption is computed based on the electrochemical reaction and the fuel cell efficiency, using standard fuel-cell relations involving Faraday’s constant and the stack operating point. In the control-oriented setting adopted here, the hydrogen consumption rate ${\dot{m}}_{H_{2}}$ is determined from the electrical output and the instantaneous efficiency (or equivalently from reaction stoichiometry), and then discretized for simulation and MPC prediction. This modeling choice is consistent with widely adopted control-oriented PEMFC modeling approaches reported in the literature, where efficiency curves are derived from hydrogen mass flow rate and lower heating value (LHV), and calibrated to bound deviations from nominal performance [36,56,57].

The hydrogen mass consumption rate is calculated as:

(12) $${\dot{m}}_{H_{2}}\left(k\right)=\frac{P_{FC}(k)}{\eta_{FC}(k){LHV}_{H_{2}}}$$

where ${\dot{m}}_{H_{2}}$(k) (kg/s) denotes the hydrogen mass consumption rate, $P_{FC}\left(k\right)\;$(W) represents the fuel cell output power, $\eta_{FC}(k)$ denotes the instantaneous fuel cell efficiency, and ${LHV}_{H_{2}}$ is the lower heating value of hydrogen. It should be noted that the hydrogen consumption is computed directly on a mass basis from the electrical output, the instantaneous fuel-cell efficiency, and the lower heating value (Equations (12) and (13)), without invoking any gas equation of state. Consequently, no ideal-gas (or real-gas) assumption enters the consumption calculation: the hydrogen lower heating value is treated as a fixed mass-specific property (${LHV}_{H_{2}}$ = 1.20 × 10^8^ J/kg), so that the reported cumulative hydrogen mass and consumption rate are independent of any assumption regarding the thermodynamic state of the gas. The instantaneous fuel-cell efficiency $\eta_{FC}(k)$ is not prescribed as a constant value; it is computed at each step directly from the operating point of the polarization model. Specifically, it is obtained as the ratio of the actual cell voltage to the thermodynamic (ideal) cell voltage of the hydrogen reaction, $\eta_{FC}(k)$ = $V_{cell}(k)$/$E_{th}$, where $V_{cell}(k)$ = $N_{cell}$ is the per-cell voltage given by the polarization model (Equation (9)) and $E_{th}$ ≈ 1.25 V is the lower-heating-value-based thermoneutral cell voltage. Because the cell voltage decreases as the drawn current increases (Figure 2), $\eta_{FC}(k)$ varies with the fuel-cell operating point throughout the mission, which is why it is treated as an instantaneous, rather than constant, quantity in Equations (12) and (13).

For the discrete-time MPC implementation, the cumulative hydrogen consumption is updated as:

(13) $$m_{H_{2}}\left(k+1\right)=m_{H_{2}}\left(k\right)+{\dot{m}}_{H_{2}}\left(k\right)∆t$$

where

$P_{FC}\left(k\right):$ fuel cell output power (W)

$\eta_{FC}(k)$: instantaneous fuel cell efficiency

${LHV}_{H_{2}}$: lower heating value of hydrogen (J/kg)

Δ*t*: sampling time s

$m_{H_{2}}\left(k\right)$: cumulative hydrogen consumption (kg)

### 3.3. Li-Po Battery Model

This subsection presents the mathematical model of the Li-Po battery subsystem integrated into the proposed PEMFC–battery hybrid UAV architecture. The battery model is developed to capture the electrical behavior and energy storage dynamics required for predictive energy management and transient load support. In the hybrid configuration, the battery plays a critical role in compensating rapid power fluctuations and supporting the PEMFC during highly dynamic operating conditions. Accordingly, the adopted formulation includes both terminal voltage behavior and SoC evolution, enabling the MPC framework to enforce operational constraints while maintaining stable and endurance-oriented system operation throughout the mission profile.

#### 3.3.1. Battery Terminal Voltage

The Li-Po battery is represented by a Thevenin equivalent circuit to capture transient voltage response and internal losses. This model structure is widely used in hybrid power system simulation and is suitable for MPC because it provides a compact state description while maintaining adequate dynamic fidelity [39,58,59].

In general form, the terminal voltage can be expressed as

(14) $$V_{bat}\left(k\right)=V_{oc}\left(SoC\left(k\right)\right)-R_{0}I_{bat}\left(k\right)-V_{RC}(k)$$

where $V_{OC}$ is the open-circuit voltage map, $V_{0}$ is internal resistance, and $V_{RC}$ represents polarization dynamics from the *RC* branch [39,58,59].

#### 3.3.2. SoC Dynamics (Coulomb Counting)

The state-of-charge is updated via coulomb counting in discrete time [58,59]:

(15) $$SoC\left(k+1\right)=SoC\left(k\right)-\frac{\eta_{bat}\Delta t\;}{C_{bat}}I_{bat}(k)$$

where $C_{bat}$ is the nominal capacity, Δ*t* is the sampling interval, and $\eta_{bat}\;$ denotes coulombic efficiency, which may differ between charging and discharging. This representation is consistent with the SoC formulation used in the prior work and supports constraint enforcement in the MPC optimization (e.g., ${SoC}_{min}\leq SoC\leq {SoC}_{max}$) [58,59].

### 3.4. Load Power Representation

The quadrotor electrical load $P_{load}$ is generated by the propulsion subsystem and varies significantly with flight phase (takeoff, climb, hover, cruise, and aggressive maneuvering) as well as external disturbances such as wind. In the proposed framework, $P_{load}(k)$ is treated as a time-varying demand profile supplied to the EMS/MPC layer, enabling evaluation of power split performance under realistic transient conditions. This modeling choice is consistent with system-level hybrid UAV modeling perspectives commonly adopted in the PEMFC-powered UAV literature [6,41,42].

#### 3.4.1. Summary of Implemented Models

The preceding subsections reviewed several candidate modeling approaches reported in the literature for each component of the hybrid powertrain. To remove any ambiguity regarding which formulations were ultimately implemented in the reported simulations and analyses, the specific models actually used in this study are consolidated in Table 2, together with the corresponding governing equations. All simulation and energy-management results presented in Section 4 are based exclusively on these implemented models; the additional formulations discussed earlier are included only to motivate and contextualize these choices and were not themselves used to generate the reported results.

#### 3.4.2. Summary of Sub-Model Parameters

To enhance the reproducibility of the proposed model, the key parameters of the individual sub-models are consolidated in Table 3. The PEMFC parameters correspond to the commercial Aerostak A-500 (500 W) air-cooled stack and are taken directly from the manufacturer datasheet, while the battery parameters correspond to the 4S LiPo pack adopted in the simulation. The values of the battery internal resistance, coulombic efficiency, and state-of-charge operating limits are not provided by the manufacturer and were therefore set to typical values reported for high-discharge-rate LiPo cells; these are indicated accordingly in the table.

Regarding the power-electronic interface, the DC–DC converter linking the fuel cell and battery to the common DC bus is represented as an ideal, lossless stage with unity efficiency ($\eta_{DC-DC}$ = 1.0). This assumption is adopted because the present study addresses supervisory, system-level energy management rather than converter-level dynamics, and it provides a conservative upper bound on the power available at the DC bus. Accordingly, converter switching and conduction losses are not modeled explicitly; their inclusion through a load-dependent efficiency map is identified as a refinement for future work. Table 3 summarizes the key parameters of the individual sub-models used in the simulation study, including the PEMFC stack, the battery, the DC–DC interface, and the MPC sampling settings.

### 3.5. MPC-Based Energy Management Problem Formulation

In the proposed PEMFC–battery hybrid quadcopter architecture, MPC is employed as the supervisory energy management strategy to coordinate power sharing between the fuel cell and the battery under dynamically varying mission conditions. Unlike conventional rule-based approaches, the considered UAV platform is subjected to highly transient propulsion load variations caused by takeoff, climb, maneuvering operation, and stochastic wind disturbances. Under such conditions, maintaining stable DC-bus power delivery while simultaneously regulating hydrogen utilization and battery SoC becomes a coupled constrained optimization problem rather than a simple power distribution task. For this reason, MPC provides an appropriate framework because it enables the explicit incorporation of system dynamics, operational constraints, and multi-objective energy management objectives within a unified predictive control formulation [19,21,60].

At each discrete sampling instant (k), the controller solves a finite-horizon optimization problem over the prediction horizon $N_{p}$ using the measured system states as initial conditions. In the adopted formulation, the fuel cell electrical power output $P_{FC}(k)$ is selected as the primary control variable, whereas the battery power is determined implicitly through the DC-bus power balance relationship. This formulation reduces the dimensionality of the optimization problem and improves computational tractability, which is particularly important for real-time implementation in multirotor UAV platforms with limited onboard computational resources [36,61].

Within the proposed hybrid UAV framework, the MPC controller seeks to satisfy multiple coupled operational objectives simultaneously. These include maintaining instantaneous power balance at the DC bus during rapidly varying mission phases, regulating battery SoC within safe operating limits to avoid excessive depletion, limiting abrupt PEMFC power variations associated with electrochemical stress, and reducing unnecessary battery current oscillations that may accelerate degradation. These objectives are incorporated into the predictive optimization framework through the cost function formulation described in the following subsection [39,61].

#### 3.5.1. Objective Function

The MPC objective function is designed to balance multiple, and potentially conflicting, performance goals, including power tracking accuracy, SoC regulation, and mitigation of component stress. Accordingly, the cost function minimized at each control step is formulated as [36,38,39]:

(16) $$J=\sum_{i=k}^{k+N_{p}-1}({w_{p}(P_{FC}\left(i\right)+P_{bat}\left(i\right)-P_{load}\left(i\right))}^{2}+w_{SoC}{\left(SoC\left(i\right)-{SoC}_{ref}\right)}^{2}+w_{bat}I_{bat}^{2}\left(i\right)+w_{FC}{∆P}_{FC}^{2}(i))$$

where $P_{load}$ denotes the electrical load demand at the DC bus, ${SoC}_{ref}$ represents the desired battery state-of-charge reference, $I_{bat}$ is the battery current, and ${∆P}_{FC}$ corresponds to the rate of change in the fuel cell power. The weighting coefficients $w_{P},\;w_{SoC},\;w_{bat}\;$ and $w_{FC}$ are non-negative scalars that reflect the relative importance of power tracking performance, SoC regulation, battery stress reduction, and fuel cell ramp-rate limitation, respectively [36,38,39,61]. Regarding the admissible values of these weights, they are non-negative scalars (*w* ≥ 0) that encode only the relative importance of the competing objectives, and they are not subject to any normalization constraint; in particular, their sum is not required to equal unity. Because the cost function in Equation (16) is minimized, multiplying all weights by a common positive factor leaves the optimal solution unchanged, so only their ratios are meaningful. For this reason the power-tracking weight is fixed to $w_{P}$ = 1.00 as a reference, and the remaining weights are expressed relative to it. Finally, the weights are held constant throughout the entire mission and are not varied dynamically as a function of the flight phase; the use of flight-situation-dependent (adaptive) weighting is identified as a direction for future work.

The weighting coefficients used in the objective function were determined through iterative sensitivity-oriented tuning trials conducted under representative UAV mission conditions. During the tuning process, different weighting combinations were evaluated in terms of DC-bus power balance performance, hydrogen utilization, battery SoC regulation, PEMFC power smoothness, and transient battery current behavior. The power tracking term $w_{P}$ was assigned the highest priority to guarantee continuous load supply during dynamically varying flight phases. The SoC regulation term $w_{SoC}$ was selected to prevent excessive battery depletion, while the battery current penalty term $w_{bat}$ and PEMFC ramp-rate penalty term $w_{FC}$ were introduced to reduce abrupt transient electrical stress and improve operational smoothness of the hybrid power system. The final weighting coefficients were selected as a compromise between power continuity, component stress mitigation, operational stability, and computational tractability within the considered mission profile [36,38,39,61].

The power tracking term enforces DC-bus power balance and minimizes load mismatch, ensuring uninterrupted operation of the propulsion and onboard systems. The SoC regulation term prevents excessive battery depletion or overcharging, while the battery current penalty limits high current excursions that may accelerate battery degradation. In addition, Penalizing rapid variations in fuel cell power mitigates degradation mechanisms such as oxygen starvation and membrane stress, while limiting battery current reduces thermal and electrochemical aging effects. These terms collectively act as degradation proxies, enabling health-conscious operation without requiring an explicit aging model [36,38,39,62].

Through this formulation, the MPC achieves a systematic trade-off between endurance maximization and component health preservation under dynamic flight conditions. Unlike conventional rule-based or heuristic energy management strategies that rely on predefined thresholds and often induce abrupt switching between power sources, the predictive and optimization-based nature of MPC enables smooth and continuous power sharing based on system dynamics and future load evolution [36,38]. As a result, component stress is proactively managed rather than reactively addressed, making the proposed MPC framework particularly well-suited for hybrid PEMFC-powered quadcopter platforms operating under highly transient flight profiles [39,61].

#### 3.5.2. Battery SoC Reference Strategy

Unlike conventional charge-sustaining strategies that enforce a fixed SoC reference, a charge-depleting SoC reference is adopted in this study. This choice is motivated by the mission-oriented nature of UAV applications, where the primary objective is to maximize flight endurance rather than preserve battery energy for indefinite operation. By allowing the battery SoC to gradually decrease within predefined safety limits, the PEMFC can operate more steadily, reducing transient stress and improving overall system efficiency [30,36,63].

The SoC reference trajectory is therefore selected within a bounded interval $[{SoC}_{min}$, ${SoC}_{max}]$, ensuring that the battery remains within a safe and functional operating region throughout the mission. This formulation provides a practical compromise between endurance maximization and battery health preservation [39].

The MPC optimization problem explicitly incorporates a set of operational constraints reflecting the physical and safety limitations of the hybrid power system:

$$\;P_{FC,min}\leq P_{FC}\left(i\right)\leq P_{FC,max},$$

$${SoC}_{min}\leq SoC(i)\leq {SoC}_{max},$$

$$P_{bat,min}\leq P_{bat}(i)\leq P_{bat,max},$$

$$\;\left|{∆P}_{FC}(i)\right|\leq P_{FC,max}$$

These constraints ensure safe operation of both the fuel cell and the battery, while preventing excessive current draw, overcharging, deep discharging, and abrupt fuel cell power ramps. By embedding these limits directly into the optimization problem, constraint satisfaction is guaranteed by design rather than enforced through heuristic post-processing [29,39,58].

At each sampling instant, the finite-horizon optimization problem was solved using an in-house MATLAB/Simulink implementation developed for the proposed hybrid UAV energy management framework. The controller evaluates admissible PEMFC power trajectories within the defined operational constraints over the prediction horizon and selects the control sequence minimizing the objective function. The adopted implementation follows a constrained finite-horizon search strategy suitable for the relatively low-dimensional control problem considered in this study. No artificial intelligence-based optimizer or external commercial MPC solver was employed. This implementation was selected to maintain transparency, computational simplicity, and direct controllability of the optimization procedure within the considered UAV energy management application.

The upper and lower operational limits defined within the MPC framework are determined by taking into account manufacturer data, electrochemical constraints, and operating ranges proposed in the literature to ensure the safe and efficient operation of the fuel cell. Particularly in PEMFC-based UAV systems, the limited power density and slow dynamic response characteristics of fuel cells make it difficult to meet sudden power demands on their own, thus necessitating the use of hybrid energy systems [64,65]. In the present work, this hybrid energy system is not treated only as a general concept from the literature but is explicitly modeled: the specific configuration considered and simulated here is a PEMFC–battery hybrid architecture, in which the PEMFC is described by the semi-empirical polarization model (Section 3.2), the battery by a Thevenin equivalent circuit with coulomb-counting state of charge (Section 3.3), and the two sources are coupled through the DC-bus power balance together with the balance-of-plant loads (Section 3.1). The operational limits discussed here are precisely the constraints imposed on this modeled hybrid system within the MPC formulation.

In this context, MPC offers an effective approach for the management of fuel cell-based UAV energy systems with complex and constrained operating conditions due to its ability to directly incorporate constraints such as maximum power, power ramp rate, and system dynamics into the model. Determining the power limits in PEMFC-based UAV systems is critical to prevent operating conditions that shorten cell life, such as overloading and high current density. The literature indicates that continuous operation in high-power regions can adversely affect fuel cell performance and accelerate degradation processes. Furthermore, it is stated that commercial PEMFC UAV systems generally prefer more simplified operating condition management systems, and therefore these systems are more sensitive to external factors such as ambient temperature, pressure, and humidity. This situation increases the importance of appropriate constraint definitions and advanced energy management strategies, particularly highlighting MPC-based approaches [65].

Similarly, the maximum battery current and PEMFC power ramp-rate limits have been determined by considering the transient response characteristics of the hybrid power system, the durability of the electrochemical components, and long-term operational reliability. The literature indicates that PEMFC systems are subject to different degradation mechanisms, especially under start/stop, idle, nominal load, and high-power operating conditions, and that dynamic load variations have a decisive effect on system lifetime [66]. During rapid load changes, short-term reactant starvation due to reactant transport limitations within the PEMFC, along with temperature and humidity gradients and sudden voltage fluctuations, can occur. This situation accelerates performance loss by creating additional stresses on the internal water/thermal distribution, catalyst layer stability, and membrane integrity. It has been reported that sudden load increases, especially under high current density, cause effects such as cathode catalyst layer degradation, Pt agglomeration, membrane fatigue, and reduction in the electrochemical active surface area [67]. It has been shown that voltage drop, instability during load transients, and catalyst layer damage become more pronounced as the ramp rate increases, whereas more controlled load transients can reduce PEMFC degradation. Additionally, it is stated that short-term reactant starvation and flooding effects occurring during dynamic load cycles accelerate irreversible damage mechanisms such as carbon corrosion, catalyst dissolution, and membrane degradation [66]. On the battery side, safe current limits have been defined considering that high current draws may increase thermal stress and aging effects. The operational limits determined in this context have been selected to limit sudden load changes on the PEMFC, reduce battery current stress, and maintain the safe operating region of the system components. Thus, the MPC-based energy management structure has been configured as a multi-objective framework that limits not only hydrogen consumption and SoC regulation but also rapid power changes that could accelerate PEMFC degradation.

Accordingly, the numerical constraint values adopted in this study were selected to remain within the manufacturer-recommended operating region of the Aerostak A-500 PEMFC stack and the safe operating limits of the Li-Po battery system. In particular, the PEMFC maximum power limit was restricted to the nominal rated operating range of the stack, the minimum battery SoC threshold was selected to avoid excessive deep discharge conditions, and the PEMFC ramp-rate constraint was chosen to reduce abrupt transient loading associated with electrochemical and thermal stress. The selected limits therefore represent a compromise between power availability, component protection, degradation mitigation, and real-time operational stability under representative UAV mission conditions.

To ensure robust convergence and real-time feasibility, the MPC optimization problem is solved using a brute-force search over a discretized set of admissible fuel cell power levels. Although this approach is computationally simple, it provides a global optimum within the predefined candidate set and avoids issues related to non-convexity and local minima that may arise in nonlinear hybrid system models. The prediction horizon Np is selected to balance control performance and computational burden, capturing the dominant dynamics of the fuel cell–battery system while remaining compatible with real-time execution constraints. Overall, this MPC formulation provides a systematic and transparent framework for hybrid energy management in PEMFC-powered quadcopters, enabling efficient power allocation, constraint satisfaction, and endurance-oriented operation under realistic flight conditions [36,38,68].

From a computational perspective, the proposed MPC framework maintains relatively low algorithmic complexity because only a single control variable, namely the fuel cell power reference, is optimized over a finite discretized candidate set at each sampling instant. Let Nc denote the number of admissible candidate control inputs and Np represent the prediction horizon length. Under the adopted brute-force search formulation, the computational complexity scales approximately with O(Nc × Np), which remains computationally tractable for the prediction horizon and discretization levels considered in this study. Owing to the low-dimensional structure of the optimization problem, the proposed formulation is considered compatible with real-time embedded implementation constraints of UAV-class onboard processors. Nevertheless, detailed execution-time benchmarking and hardware-level latency analysis on practical embedded platforms remain important topics for future hardware-in-the-loop validation studies.

To further clarify the practical deployability of the proposed strategy on resource-constrained UAV platforms, the model-simplification choices, the rationale behind the prediction-horizon selection, the optimization solver, and an analytical real-time feasibility assessment for typical onboard processors are detailed below.

**Model simplification.** The control-oriented model deliberately replaces high-fidelity, spatially distributed electrochemical and multi-physics PEMFC representations with a lumped semi-empirical polarization model (Equations (9)–(11)), augmented by a single first-order transient voltage term (Equations (3) and (4)) and a coulomb-counting battery model (Equations (14) and (15)). This reduces the predicted system state to a low-dimensional set, namely the battery SoC, the filtered PEMFC operating point, and the DC-bus power balance, thereby eliminating the stiff partial-differential dynamics and large state vectors that render detailed electrochemical models computationally prohibitive for embedded prediction. The adopted simplification preserves the dominant low-frequency dynamics that govern power sharing and endurance, which are precisely the quantities the supervisory controller must regulate, while the high-frequency transient component is delegated to the fast-response battery buffer.

**Horizon and discretization selection.** A sampling time of Δt = 1 s and a prediction horizon of Np = 10 steps, corresponding to a 10 s look-ahead, were selected to match the dominant time scales of the supervisory energy-management problem rather than the fast electrical transients, which are buffered by the battery. The 1 s sampling interval is consistent with the slow PEMFC dynamics (effective time constant on the order of seconds) and with the ramp-rate-limited fuel-cell power command (${∆P}_{FC,max}$ = 20 W/s); a finer interval would therefore yield no additional control authority over the fuel cell while increasing the computational load. The 10-step horizon is sufficiently long to anticipate battery SoC drift and mission-phase transitions, yet short enough to keep the candidate search space small. During tuning, horizons beyond this value produced negligible changes in endurance and power-continuity metrics while increasing computation approximately linearly. No separate control horizon is employed, since a single control input, namely the PEMFC power reference, is optimized at each sampling instant.

**Optimization solver.** At each sampling instant, the controller performs an exhaustive (brute-force) evaluation over a discretized candidate set of admissible PEMFC power levels. The admissible range [$P_{FC,min},\;P_{FC,max}$] is discretized at a fixed resolution, yielding Nc candidates; for the present configuration (0–500 W at a 5 W resolution), this corresponds to Nc ≈ 100 candidates. Each candidate is propagated over the Np-step horizon and scored using the cost function in Equation (16), subject to the operational constraints defined in Section 3.5.2. This procedure guarantees the global optimum within the candidate set and avoids the convergence, initialization, and local-minimum issues associated with gradient-based or interior-point solvers applied to the non-convex hybrid model, at the cost of a deterministic and bounded computational budget.

**Real-time feasibility analysis.** The per-step computational cost scales as O(Nc × Np). For the adopted configuration (Nc ≈ 100, Np = 10), this amounts to on the order of 10^3^ candidate-step evaluations per control update, each consisting of a small number of algebraic floating-point operations from the lumped model. This workload is several orders of magnitude below the throughput of modern UAV-class flight-control processors. Typical autopilot platforms are built on ARM Cortex-M-class microcontrollers equipped with hardware floating-point units operating at hundreds of MHz (e.g., the STM32H7-based controllers used in Pixhawk-class autopilots), which sustain tens to hundreds of millions of floating-point operations per second. Consequently, the estimated worst-case execution time per control update remains a small fraction of the 1 s sampling period, leaving a substantial real-time margin for the sensing, state-estimation, and flight-control tasks that run concurrently. Comparable control-oriented predictive energy-management formulations have been reported to execute within embedded real-time constraints on similar hardware, which supports the feasibility of the proposed strategy [36,45,61]. It should nonetheless be emphasized that the present assessment is analytical; direct execution-time benchmarking and hardware-in-the-loop latency measurement on a target embedded platform remain part of the planned validation work described in Section 5.

In the present implementation, the MPC framework does not incorporate an explicit disturbance forecasting or wind prediction module within the prediction horizon. Instead, stochastic wind disturbances are introduced as external time-varying load perturbations affecting the propulsion power demand, and the controller reacts to these variations based on the updated system states at each sampling instant. Therefore, the proposed formulation should be interpreted as a disturbance-reactive MPC strategy rather than a disturbance-predictive framework. Incorporating mission-aware load forecasting and wind prediction mechanisms into the predictive layer represents an important direction for future research.

In this study, health awareness is incorporated in an implicit manner rather than through an explicit state-of-health (SoH) model. Specifically, degradation-related effects are approximated by penalizing fuel cell power ramp rates and battery current levels within the MPC cost function. These quantities are strongly correlated with known degradation mechanisms such as membrane stress, catalyst degradation, and battery aging. Therefore, the proposed formulation can be interpreted as an implicit degradation-aware stress-mitigation strategy, where component stress is regulated through degradation-related proxy variables rather than through direct electrochemical aging estimation.

It should be noted that the degradation-aware capability considered in this study is implemented in an implicit and control-oriented manner through stress-related proxy terms, namely fuel cell power ramp-rate limitation and battery current penalization. Although these variables are closely associated with known degradation mechanisms such as membrane stress, catalyst aging, and electrochemical loading effects, the proposed framework does not include a detailed electrochemical aging model or reversible voltage recovery dynamics. In practical PEMFC systems, degradation behavior is governed by complex coupled physicochemical mechanisms that may exhibit both reversible and irreversible characteristics over long-term operation. Therefore, the present formulation should be interpreted as a computationally efficient stress-mitigation strategy rather than a full physics-based health prediction framework. Incorporating higher-fidelity electrochemical degradation and reversible aging models into the predictive control architecture remains an important direction for future research.

#### 3.5.3. MPC Parameter Selection

The main MPC controller parameters used in the simulations are summarized in Table 4. The prediction horizon and weighting coefficients were experimentally selected to balance power tracking performance, battery SoC regulation, reduction in fuel cell stress, and real-time computational feasibility. In particular, the PEMFC power ramp-rate constraint was conservatively determined to prevent excessive transient loading and oxygen starvation effects during aggressive maneuvering conditions.

**Figure 3:**
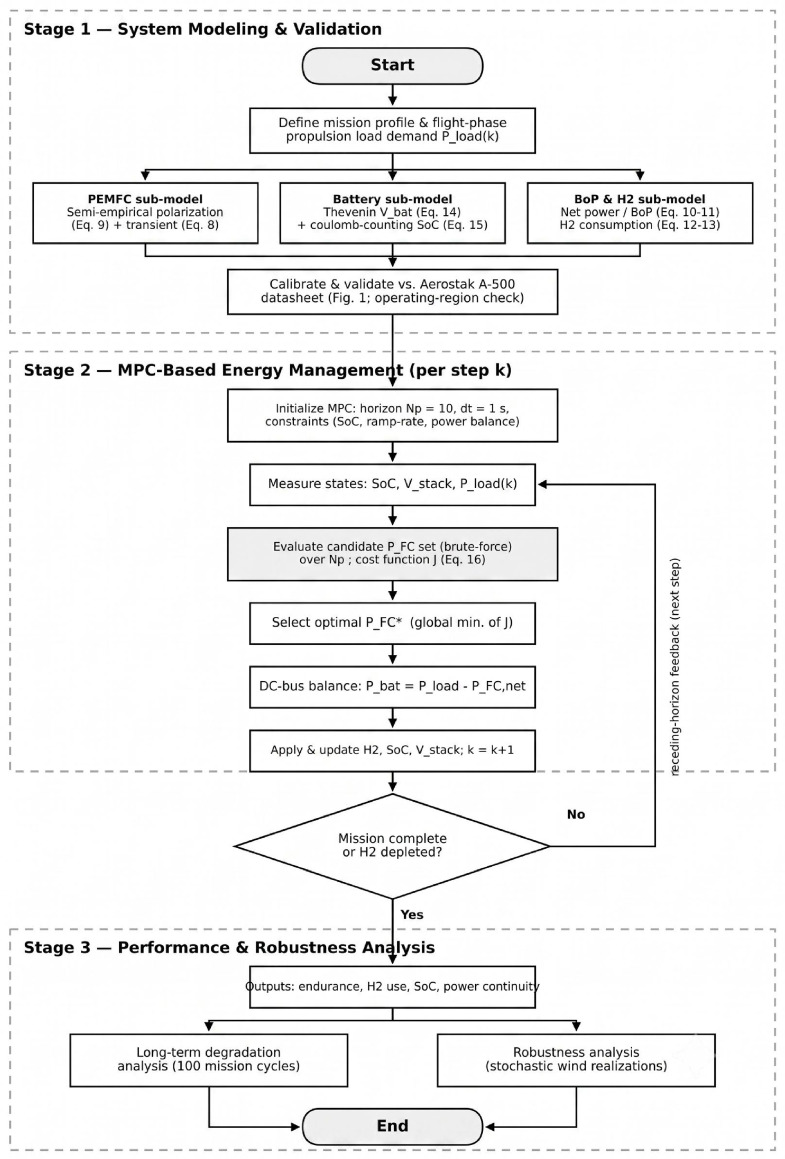
Overall methodology and computational workflow of the proposed MPC-based energy management framework, comprising system modeling and validation (Stage 1), the per-step receding-horizon MPC optimization loop (Stage 2), and the performance and robustness analyses (Stage 3).

To summarize the overall methodology and computational workflow of the study, Figure 3 presents a flowchart of the complete process, from system modeling and validation, through the per-step MPC-based energy-management optimization, to the final performance and robustness analyses.

## 4. Simulation Results and Discussion

The proposed MPC-based energy management framework was validated using the high-fidelity hybrid UAV model described in Section 3. The simulation scenarios were designed to evaluate system performance under representative multirotor UAV operating conditions, including dynamic mission-phase transitions and stochastic environmental disturbances. To this end, a mission-based load profile was constructed to emulate typical quadcopter flight phases, namely takeoff, climb, hover, cruise, and aggressive maneuvering operation. Each phase was associated with different propulsion power demand levels reflecting the varying aerodynamic and thrust requirements encountered during UAV operation.

In particular, aggressive maneuvering phases were modeled using rapidly varying transient load demands to represent abrupt changes in vehicle orientation, acceleration, and flight speed. To further evaluate controller robustness under realistic environmental conditions, stochastic wind disturbances with a mean wind speed of approximately $v_{mean}$ = 4 m/s and additional random gust components were superimposed on the nominal propulsion load profile as time-varying disturbance signals. These disturbances emulate short-term turbulence-induced fluctuations in propulsion power demand and external aerodynamic perturbations affecting UAV stability and power consumption.

The resulting composite load profile was applied as an external load input to the PEMFC–battery hybrid power system throughout the simulation duration and used to evaluate the robustness and energy management capability of the proposed MPC framework under dynamically varying operating conditions.

### 4.1. Power Distribution and Dynamic Response

**Figure 4:**
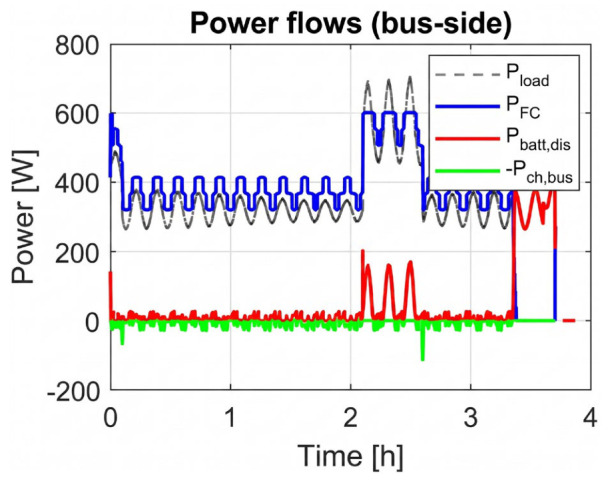
Power distribution among the load, PEMFC, and battery on the DC bus side.

The fundamental capability of the EMS to coordinate the hybrid power sources is illustrated in Figure 4 The load profile (black dashed line) exhibits significant fluctuations due to the turbulent wind model and aggressive maneuvering phases, particularly between *t* = 2.1 h and *t* = 2.6 h.

**Figure 5:**
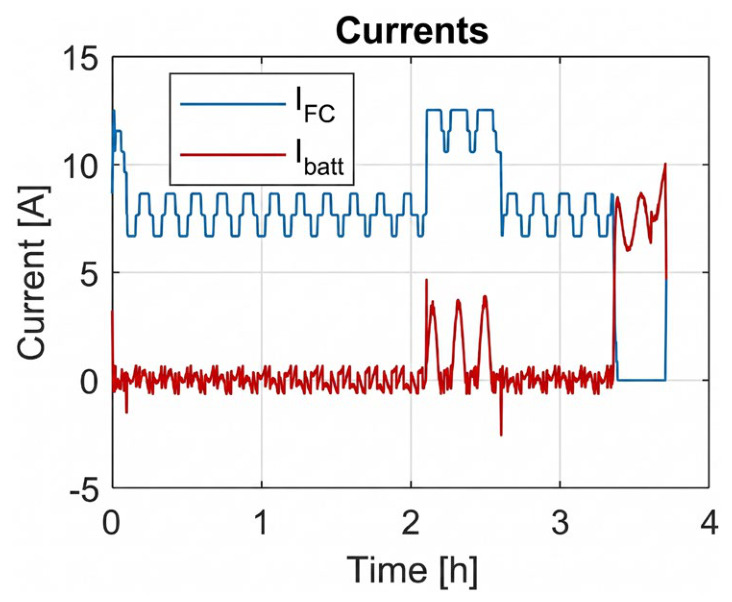
Current profiles of the PEMFC and Battery, highlighting the buffering effect of the battery during transients.

As observed in Figure 4 and further detailed in Figure 5 (Currents), the proposed MPC-based energy management strategy effectively decouples the fuel cell dynamics from high-frequency load variations. The PEMFC predominantly follows the low-frequency component of the power demand, while its power ramp rate is explicitly constrained, resulting in smooth and gradual power transitions even under abrupt load changes. This controlled behavior is particularly critical for mitigating oxygen starvation and reducing electrochemical stress, thereby contributing to prolonged fuel cell stack lifetime. In contrast, the battery operates as a fast-response energy buffer, instantaneously supplying power during transient peaks such as those occurring in the agile maneuver phase and absorbing excess energy during low-load intervals. Compared to conventional rule-based strategies, which often suffer from abrupt switching or chattering effects, the MPC framework enables a continuous and optimally blended power sharing between the fuel cell and the battery, ensuring both power continuity and component-friendly operation.

Within the simulation framework, the PEMFC model, Li-Po battery model, DC-bus power balance equations, mission-phase load profile, and MPC controller were integrated into a unified closed-loop hybrid UAV energy management architecture implemented in MATLAB/Simulink. At each sampling instant, the mission-dependent propulsion load demand and stochastic disturbance signals were applied to the DC bus as external power requests. The MPC controller then received the updated system states, including battery SoC, PEMFC operating conditions, and load demand information, and computed the optimal PEMFC power reference subject to the defined operational constraints and cost-function objectives.

The battery power contribution was subsequently determined through the DC-bus power balance relationship, while the PEMFC and battery dynamic models updated their internal states for the next prediction interval. In this manner, the electrochemical PEMFC behavior, battery dynamics, transient load variations, and predictive control strategy interacted continuously within a unified time-domain simulation environment throughout the mission profile.

### 4.2. Electrochemical Behavior and Voltage Stability

**Figure 6:**
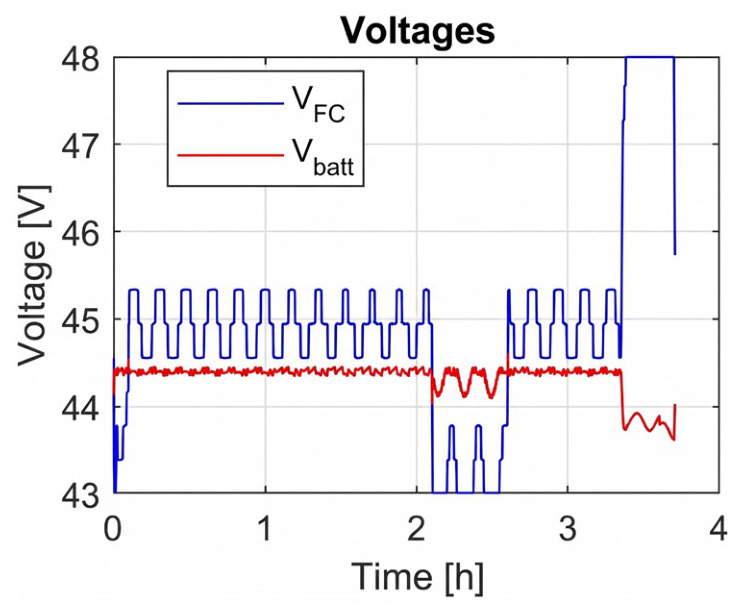
Voltage response of the hybrid sources. The PEMFC voltage exhibits realistic polarization drops proportional to load current.

To verify the physical consistency of the simulation, the voltage and thermal characteristics of the power sources were analyzed. Figure 6 presents the voltage response of the PEMFC and the battery.

The PEMFC voltage $V_{FC}$ exhibits a realistic inverse correlation with the load current. During high-power demand phases (Agile Phase), the voltage drops from the open-circuit value (~48 V) to approximately 43 V due to ohmic and activation losses, as predicted by the electrochemical model. This confirms that the MPC is optimizing the system based on a realistic plant model rather than an idealized constant-voltage source.

**Figure 7:**
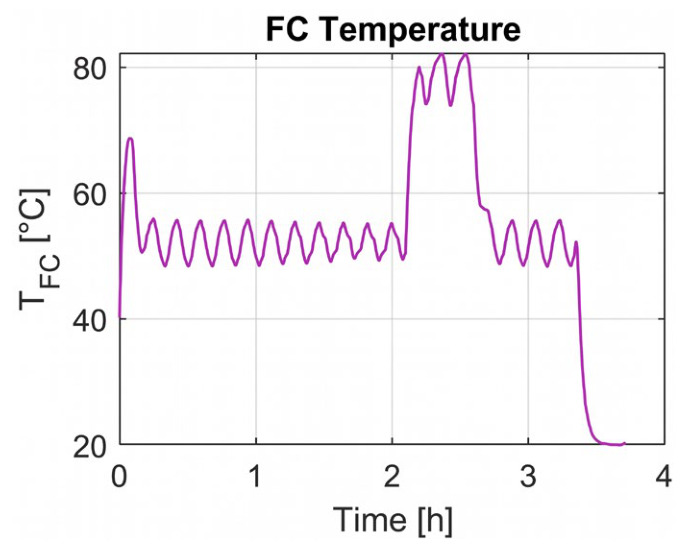
PEMFC stack temperature evolution during the mission.

Furthermore, the thermal response of the stack is shown in Figure 7. The temperature rises significantly during the high-load agile phase (*t* ≈ 2.2 h), reaching a peak of approximately 80 °C. This value remains within the permissible thermal limits of the modeled Aerostak unit, indicating that the EMS implicitly manages the thermal load by preventing prolonged overload conditions.

### 4.3. Battery SoC Management and Strategic Optimization

**Figure 8:**
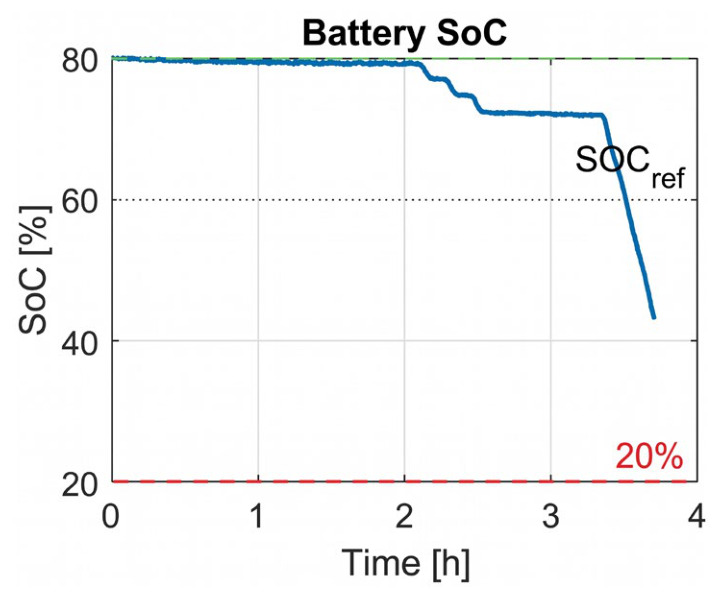
Battery SoC evolution compared to the charge-depleting reference trajectory.

A critical feature of the proposed MPC is its ability to balance immediate constraints with long-term objectives. Figure 8 illustrates the Battery SoC evolution against the reference trajectory. A “Charge-Depleting” reference strategy was fed to the controller (black dotted line), theoretically guiding the battery to discharge linearly from 80% to 25%.

**Figure 9:**
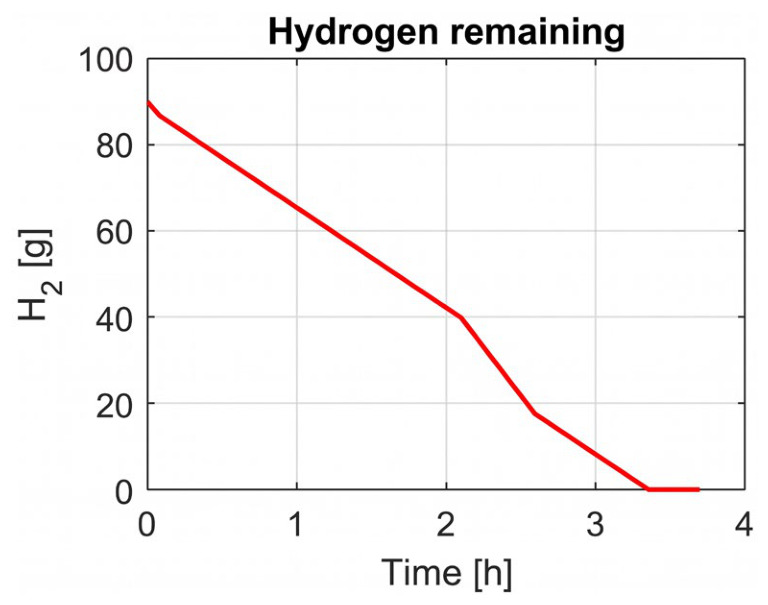
Hydrogen fuel consumption profile. The depletion point triggers the battery discharge phase.

However, a notable behavior is observed in the actual SoC profile (blue line):

- **Safety-Prioritized Deviation:** The MPC algorithm autonomously deviates from the strict reference trajectory, maintaining the SoC at a high level (~75–80%) for the majority of the flight (0 to 3.2 h).
- **Optimization Logic:** The cost function J weighs the penalty of potential unmet power heavily. Consequently, the controller “decides” to conserve battery energy as a safety reserve against stochastic wind gusts, relying primarily on hydrogen fuel.
- **End-of-Mission Depletion:** Only when the hydrogen supply approaches depletion (after *t* = 3.2 h, as shown in Figure 9) does the controller aggressively discharge the battery to sustain the flight, successfully utilizing the remaining electrical energy to maximize endurance.

This result demonstrates that the proposed MPC is not merely a trajectory tracker but an intelligent decision-making agent that prioritizes system robustness over simple reference following.

### 4.4. Trajectory Tracking and System Robustness

**Figure 10:**
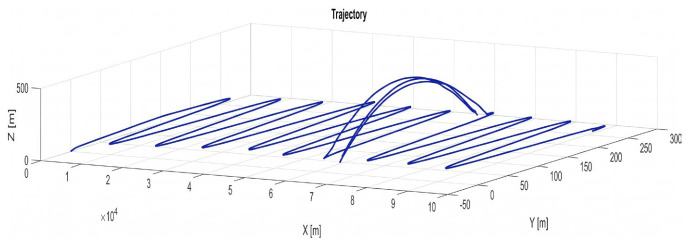
3D flight trajectory of the UAV during the simulation.

The ultimate goal of the power system is to support the flight mission. Figure 10 shows the 3D trajectory of the UAV, which successfully completed the lawnmower survey pattern including the altitude changes defined in the mission profile.

**Figure 11:**
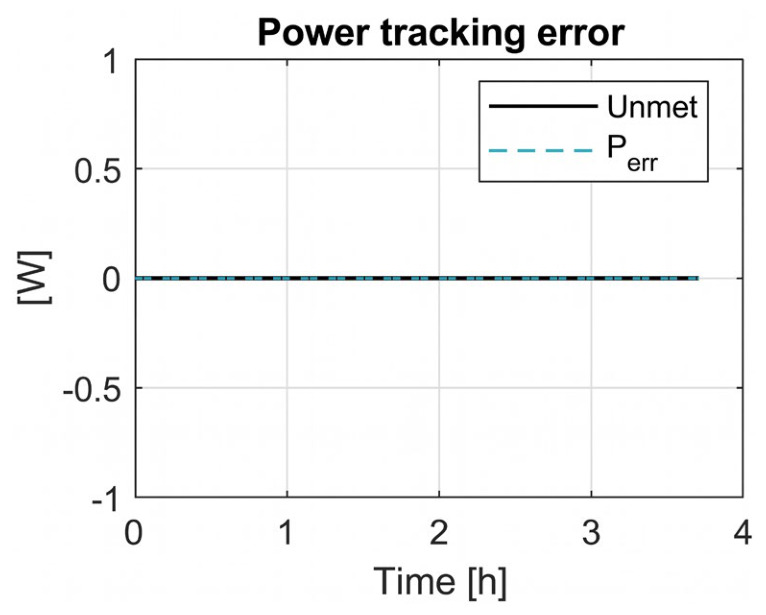
Power tracking error, confirming that load demand was fully met throughout the mission.

As evidenced by the Power Tracking Error graph in Figure 11, the unmet load power remained at zero throughout the mission. This confirms that the hybrid power system, governed by the MPC, successfully met the propulsion demands at every time step without violating voltage or current constraints.

To provide a comprehensive overview of the mission performance, the aggregate simulation results are summarized in Table 5. This table highlights the key metrics achieved by the proposed MPC-based energy management strategy, confirming zero unmet energy and efficient hydrogen utilization.

### 4.5. Performance Benchmarking

To objectively assess the effectiveness of the proposed MPC-based energy management strategy, a comparative benchmarking analysis is conducted against a representative rule-based energy management system reported in the literature for PEMFC-powered multirotor UAVs. The selected reference reflects a conventional supervisory control approach widely adopted in early hybrid UAV implementations, where power sharing is governed by predefined rules and frequency separation techniques rather than predictive optimization.

It is important to note that the baseline and proposed systems differ in terms of platform configuration, mission duration, and operating conditions. Therefore, the comparison is intended to highlight relative performance trends rather than direct numerical equivalence. The benchmarking focuses on key endurance-oriented metrics, including hydrogen consumption characteristics, power flow smoothness, and battery SoC management behavior. The comparative results are summarized in Table 6.

Table 6 presents a qualitative and trend-level comparison between a representative rule-based energy management strategy reported in the literature and the proposed MPC-based energy management framework for a PEMFC–battery hybrid multirotor UAV. The rule-based baseline is derived from Boukoberine et al. [38] and represents a typical heuristic supervisory control approach, while the MPC results correspond to the proposed system developed in this study. It should be emphasized that the compared systems differ in terms of UAV platform configuration, mission duration, load profiles, hydrogen storage capacity, payload characteristics, and environmental operating conditions. Therefore, the presented comparison is not intended to establish strict one-to-one numerical superiority or experimental validation, but rather to provide a qualitative and trend-level assessment of how different energy management strategies influence hydrogen utilization behavior, power stability, endurance characteristics, and overall energy management performance.

### 4.6. Sensitivity and Long-Term Reliability Analysis

To avoid ambiguity, the experimental settings and evaluation criteria adopted for this long-term analysis are defined explicitly as follows. In this study, a single cycle denotes one complete UAV mission profile, comprising the takeoff, climb, hover, cruise, and aggressive-maneuvering phases described in Section 4, executed until hydrogen depletion. Thus, the term “100 cycles” refers to 100 repetitions of this complete mission profile rather than to 100 isolated fuel-cell load cycles or start/stop events, which is consistent with the corresponding operational duration of approximately 350–400 h. Fuel-cell aging is modeled at the cycle level: at the start of each cycle, the stack voltage Vstack is reduced according to the adopted aging model (a constant decay of 300 µV per cell per cycle for the linear case, or the nonlinear profile described below), and this degraded voltage–current characteristic is then used by the plant model throughout that cycle. The degradation therefore enters the “degradation-aware” MPC framework implicitly through the plant: as Vstack decreases with cycle number, the controller must draw a higher stack current to satisfy the same propulsion power demand (Preq = Vstack∙Istack), which increases the per-cycle hydrogen consumption and progressively shortens the achievable flight time. The endurance-retention metric is defined as the ratio of the achievable flight time (endurance until hydrogen depletion) at the 100th cycle to that at the first (start-of-life) cycle, expressed as a percentage; the reported value of over 97% corresponds to the decrease from 220–224 min at start-of-life to approximately 215.6 min after 100 cycles. The associated rise in cumulative hydrogen consumption per mission is the underlying physical driver of this endurance reduction and is captured by the same simulation, rather than being a separate independent metric.

In practical UAV operations, the performance of PEMFC stacks inevitably degrades over time due to electrochemical and mechanical aging mechanisms such as membrane thinning, catalyst agglomeration, and carbon support corrosion. To evaluate the long-term robustness of the proposed MPC-based energy management framework, a degradation sensitivity analysis was conducted over 100 consecutive flight cycles, corresponding to approximately 350–400 operational hours. Two representative aging models were considered for the fuel cell stack voltage $V_{stack}$. The first model assumes a constant voltage decay rate of 300 µV per cycle, representing gradual ohmic resistance growth, while the second adopts a nonlinear degradation profile characterized by an initially accelerated activation loss followed by a milder aging phase, which more closely reflects experimentally observed PEMFC degradation behavior. It should be noted that these degradation profiles are introduced to assess the sensitivity of system-level performance to fuel cell aging trends, rather than to predict absolute lifetime, and are adopted here as commonly reported aging representations in the PEMFC literature [62,69,70]. It is worth noting that, beyond the fixed linear and nonlinear aging profiles adopted here for sensitivity analysis, more recent data-driven approaches have been developed to predict PEMFC performance degradation and quantify state-of-health with higher accuracy, including Transformer-based degradation-prediction models [25] and methods coupling data-driven techniques with electrochemical impedance information for degradation quantification [26]. The integration of such advanced degradation-prediction and health-estimation modules into the predictive control layer represents a promising direction for strengthening the long-term durability assessment of the proposed framework in future work.

**Figure 12:**
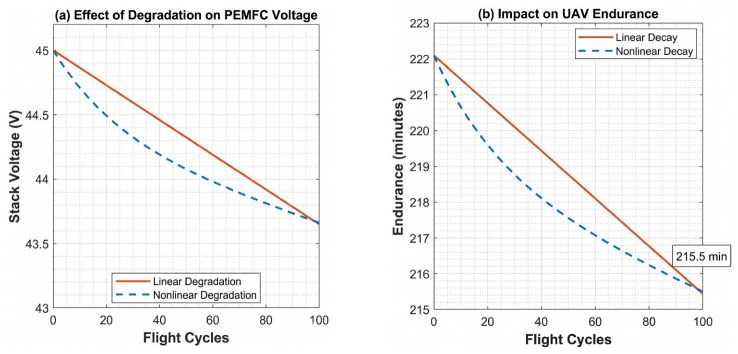
Long-term reliability analysis: (**a**) Simulated degradation of the PEMFC stack voltage over 100 flight cycles using linear and nonlinear aging models; (**b**) The corresponding impact on mission endurance. The system maintains over 97% of its initial endurance capacity after 100 cycles, demonstrating high operational robustness.

The impact of fuel cell degradation on system performance is illustrated in Figure 12. As shown in Figure 12a, the nonlinear aging model results in a cumulative stack voltage reduction of approximately 3.2% after 100 flight cycles. To maintain the required propulsion power ($P_{req}=V_{stack}I_{stack}$), the controller compensates for the reduced voltage by operating at higher current levels, which in turn increases hydrogen consumption. The corresponding impact on mission endurance is quantified in Figure 12b, where the achievable flight time decreases from 220–224 min at start-of-life to approximately 215.6 min after 100 operating cycles. Despite this gradual reduction, the MPC-based energy management strategy effectively adapts to the degraded operating conditions while maintaining stable system behavior and respecting all safety-related constraints. Particularly even under aged conditions, the achieved endurance level remains comparatively advantageous with respect to the typical endurance ranges reported in the literature for battery-only multi-rotor UAV configurations and representative rule-based hybrid energy management strategies. Nevertheless, it should be particularly emphasized that the presented results are intended to provide a system-level trend analysis performed under representative degradation assumptions, rather than constituting an experimentally validated absolute lifetime estimation. This observation indicates that the proposed MPC framework preserves a meaningful endurance margin over the effective lifetime of the fuel cell stack, thereby supporting reliable long-duration mission execution under realistic aging effects [62,69,70].

To assess the robustness of the proposed framework under stochastic disturbances, additional simulation runs were conducted using different realizations of the wind disturbance profile. The obtained results exhibited consistent system behavior in terms of power balance, hydrogen consumption trends, and SoC regulation, with only minor variations observed across runs. This indicates that the proposed MPC strategy maintains stable performance under varying environmental conditions.

It is also worth discussing the extent to which the proposed strategy depends on an accurate quadrotor power-demand model. In the adopted formulation, the total propulsion power demand Pload is supplied to the supervisory MPC layer as an externally measured or estimated time-varying signal, rather than being reconstructed internally from the detailed aerodynamic, mass, and flight-controller dynamics of the quadrotor. Consequently, the power-allocation task solved by the MPC is conditioned on the instantaneous value of Pload and does not itself depend on the fidelity of the underlying aerodynamic or mass model; any inaccuracy in the demand model manifests only through the demand signal that the controller is required to track. This architectural choice provides a degree of structural robustness to model mismatch, for two reasons. First, the battery operates as a fast-response buffer that instantaneously absorbs the difference between the demanded load and the comparatively slowly varying fuel-cell power reference, so that transient discrepancies between the assumed and actual power demand are accommodated by the battery rather than propagating into constraint violations. Second, the receding-horizon nature of the controller, which re-solves the optimization at every sampling instant using the most recent measured states, introduces an inherent feedback correction that compensates for persistent demand mismatch over successive steps. The consistent behavior observed across different stochastic wind realizations, reported above, is in line with this expected robustness, since wind acts precisely as an unmodeled perturbation of the propulsion demand. Accordingly, a moderate mismatch in the power-demand model is expected to degrade power-allocation effectiveness only gradually rather than abruptly. Nevertheless, the present framework employs a nominal (certainty-equivalence) MPC formulation that does not explicitly quantify or bound such uncertainty. The explicit incorporation of adaptive MPC, in which the demand or model parameters are estimated online, or of robust MPC techniques such as tube-based formulations that guarantee constraint satisfaction under bounded uncertainty, therefore represents a natural and planned extension of this work, and is expected to further improve allocation effectiveness and reliability under larger model mismatch.

## 5. Conclusions

This paper presented an MPC-based energy management framework for a PEMFC–battery hybrid quadcopter aimed at achieving reliable long-endurance operation under dynamic mission conditions. A physics-consistent system model was employed to capture the coupled electrical, electrochemical, and operational constraints of the hybrid powertrain, enabling the controller to allocate power smoothly between the PEMFC and the battery while maintaining DC-bus power continuity. The simulation study demonstrated that the proposed MPC strategy successfully met the propulsion demand at all time steps without unmet load power, confirming robust power balance and constraint satisfaction throughout the mission. Quantitatively, the hybrid system achieved an endurance of 220–224 min until hydrogen depletion while consuming 89.99 g of hydrogen with a mean consumption rate of 0.398–0.412 g/min, and maintained zero unmet energy over the entire flight profile. Compared with representative rule-based energy management strategies reported in the literature, the proposed MPC framework demonstrated a tendency toward lower hydrogen consumption behavior under the evaluated operating conditions. It is important to emphasize that the compared systems differ in terms of platform configuration, mission duration, load characteristics, payload conditions, and hydrogen storage capacity. Therefore, the comparison is not intended to establish strict one-to-one numerical superiority, but rather to provide a qualitative and trend-level evaluation of how different energy management strategies influence hydrogen utilization behavior, power stability, and overall endurance characteristics. From a normalized energy utilization perspective, the proposed MPC framework enabled more effective use of hydrogen by extracting a greater amount of useful electrical energy per unit fuel under the considered mission profile. Accordingly, the observed reduction in hydrogen consumption should be interpreted as a system-level performance trend rather than an absolute quantitative performance gain. Furthermore, the long-term sensitivity analysis indicated that, even under representative linear and nonlinear voltage degradation trends, the system retained over 97% of its initial endurance after 100 flight cycles, underscoring the operational robustness of the proposed framework for repeated mission execution. Additionally, repeated simulations under different stochastic disturbance realizations exhibited consistent system behavior in terms of power balance, hydrogen consumption trends, and SoC regulation, supporting the robustness of the proposed framework beyond a single operating scenario. Despite the promising system-level performance observed in the simulation environment, several limitations of the present study should be acknowledged. Although the present study focused on a high-fidelity simulation framework, future work will include validation of the proposed control architecture under more deployment-realistic conditions, including hardware-in-the-loop (HIL) implementation and/or experimental flight testing using embedded real-time platforms such as STM32- or Pixhawk-based systems. Such studies will enable detailed investigation of discretization effects, computational latency, and real-time execution feasibility. Future extensions will also focus on integrating mission-aware load forecasting, higher-fidelity degradation and thermal coupling models, and systematic cost-weight tuning strategies to further strengthen the relationship between endurance maximization, robustness, and long-term component health preservation in practical hybrid UAV systems.

## Figures and Tables

**Table 1:** Mass budget and BoP breakdown of the hybrid PEMFC-powered quadcopter.

Component	Description/Type	Mass (g)	% Total Mass
**Fuel Cell Stack**	500 W rated (air-cooled, 45 cells)	1580	27.4%
**H_2_ Storage System (BoP)**	Tank (3 L, 400 bar) + regulators + valves + tubing	1750	30.3%
**Battery Pack**	Li-Po 4S 8000 mAh (buffer & peak power)	755	13.1%
**Propulsion System**	4× BLDC motors + 4× ESCs + 4× propellers	391	6.8%
**Airframe**	Carbon fiber quadrotor frame	950	16.5%
**Power Electronics**	DC/DC converters (FC & battery paths)	240	4.2%
**Avionics & Payload**	Flight controller, GPS, telemetry, sensors	200	3.5%
**TOTAL (MTOM)**	Hybrid UAV take-off mass	5766 g	100%

Note: The hydrogen storage system corresponds to a compressed hydrogen tank configuration with a nominal storage volume of 3 L at 400 bar. The simulations assume an initial hydrogen mass of approximately 95 g stored at an operating temperature of 298 K, accounting for compressed-gas storage conditions rather than atmospheric-condition estimations.

**Table 2:** Summary of the sub-models actually implemented in the simulation study and their governing equations.

Sub-Model	Implemented Approach	Equation(s)
**PEMFC stack voltage**	Semi-empirical polarization model (open-circuit/Nernst, Butler–Volmer activation, Amphlett-based ohmic, logarithmic concentration losses), calibrated to Aerostak A-500 datasheet	Equation (9)
**PEMFC transient voltage**	First-order (time-constant) recovery term for double-layer/diffusion delays	Equation (8)
**Net FC power and BoP**	Gross power minus parasitic auxiliary (BoP) load	Equations (10) and (11)
**Hydrogen consumption**	Efficiency- and LHV-based mass-flow relation with discrete-time cumulative integration	Equations (12) and (13)
**Battery terminal voltage**	First-order Thevenin equivalent circuit (OCV, series resistance, single RC branch)	Equation (14)
**Battery SoC**	Coulomb-counting in discrete time	Equation (15)
**Propulsion load**	Externally supplied time-varying demand profile (flight-phase + wind disturbance)	–
**DC–DC interface**	Ideal, lossless stage (unity efficiency)	–
**Energy management**	Receding-horizon MPC with single control input (P_FC), brute-force search over discretized candidates	Equation (16)

**Table 3:** Key sub-model parameters of the PEMFC–battery hybrid power system used in the simulation study.

Parameter	Symbol	Value/Unit
**PEMFC stack** **—** **number of cells**	Ncell	45
**PEMFC rated power**	$P_{FC,rated}$	500 W
**PEMFC stack voltage range**	$V_{FC}$	28.0–42.8 V
**PEMFC stack current range**	$I_{FC}$	0–20 A
**Max. hydrogen consumption**	${\dot{m}}_{H_{2},max}$	<5.6 L/min
**Hydrogen inlet pressure**	$p_{H_{2}}$	0.6–0.8 bar
**Lower heating value of hydrogen**	${LHV}_{H_{2}}$	1.20 × 10^8^ J/kg (120 MJ/kg)
**Battery configuration**	–	4S (4 cells in series)
**Battery nominal voltage**	$V_{bat,nom}$	14.8 V
**Battery nominal capacity**	$C_{bat}$	8.0 Ah (8000 mAh)
**Continuous discharge rating**	–	40C (320 A)
**Peak discharge rating (10 s)**	–	80C (640 A)
**Battery internal resistance ^a^**	$R_{0}$	≈5 mΩ
**Coulombic efficiency ^a^**	$\eta_{bat}$	0.98
**SoC operating limits ^a^**	${SoC}_{min}$–${SoC}_{max}$	20–90%
**DC–DC converter efficiency ^b^**	$\eta_{DC-DC}$	1.0 (ideal, lossless)
**Sampling time**	Δt	1 s
**Prediction horizon**	Np	10 steps

^a^ Typical values assumed for high-discharge-rate LiPo cells (not provided by the manufacturer). ^b^ Modeled as an ideal lossless stage at the system level.

**Table 4:** MPC Controller Parameters.

Parameter	Symbol	Value
**Prediction horizon**	$N_{p}$	10 steps
**Sampling time**	∆t	1 s
**Initial battery SoC**	${SoC}_{0}$	80%
**Minimum battery SoC**	${SoC}_{min}$	25%
**Maximum battery SoC**	${SoC}_{max}$	80%
**PEMFC maximum power**	$P_{FC,max}$	500 W
**Battery current limit**	$I_{bat,max}$	±10 A
**PEMFC ramp-rate limit**	${∆P}_{FC,max}$	20 W/s
**Power tracking weight**	$w_{p}$	1.00
**SoC regulation weight**	$w_{SoC}$	0.25
**Battery current penalty**	$w_{I}$	0.08
**PEMFC ramp penalty**	$w_{∆p}$	0.15

**Table 5:** Performance Results of the Proposed MPC-Based Energy Management Strategy.

Metric	Value (MPC)	Unit
**Endurance until H_2_ depletion**	220–224	min
**Mean load power**	356.6	W
**Peak load power**	704.5	W
**Mean fuel cell power**	366.8	W
**Total H_2_ consumed**	89.99	g
**Mean H_2_ consumption rate**	0.398–0.412	g/min
**Battery SoC (initial → final)**	80.0 → 42.9	%
**Battery SoC range (max–min)**	37.09	%
**Battery RMS current**	2.465	A
**Battery peak current (absolute)**	10.1	A
**Battery net energy supplied to bus**	118.4	Wh
**Total unmet energy**	0	Wh
**Maximum unmet power**	0	W

**Table 6:** Trend-level comparison between representative rule-based EMS approaches and the proposed MPC framework.

Metric	Reference Rule-Based EMS (Boukoberine et al. [38])	Proposed MPC EMS (This Work)	Observed Trend
**Strategy Type**	Frequency Separation + Rule-Based	Model Predictive Control	Predictive optimization
**System Architecture**	Hybrid (PEMFC + Battery)	Hybrid (PEMFC + Battery)	Comparable
**Mission Duration**	42 min	220–224 min	Longer mission
**Total H_2_ Consumption**	22.05 g	89.99 g	Mission-dependent
**Mean H_2_ Consumption Rate**	≈0.52 g/min	0.398–0.412 g/min	Lower observed consumption tendency
**Power Stability**	Filtered but reactive	Smooth and ramp-limited	Smoother operational behavior
**Battery SoC Management**	Threshold-based	Constraint-aware regulation	Enhanced reserve
**Endurance Capability**	Limited by heuristic control	Longer endurance tendency	Improved robustness

## Data Availability

The original contributions presented in this study are included in the article. Further inquiries can be directed to the corresponding author.

## Abbreviations

The following abbreviations are used in this manuscript:

**Abbreviation**	**Definition**
PEMFC	Proton Exchange Membrane Fuel Cell
UAV	Unmanned Aerial Vehicle
MPC	Model Predictive Control
EMS	Energy Management System
SoC	State of Charge
SoH	State of Health
DC	Direct Current
BoP	Balance of Plant
MTOM	Maximum Take-Off Mass
Li-Po	Lithium Polymer Battery
BLDC	Brushless Direct Current (Motor)
ESC	Electronic Speed Controller
LHV	Lower Heating Value
MAPE	Mean Absolute Percentage Error
OCV	Open Circuit Voltage
RC	Resistor-Capacitor (Equivalent Circuit)
RMS	Root Mean Square
H_2_	Hydrogen
DC–DC Converter	Direct Current to Direct Current Converter
